# Validation of dynamic risk stratification and impact of BRAF in risk assessment of thyroid cancer, a nation-wide multicenter study

**DOI:** 10.3389/fendo.2022.1071775

**Published:** 2023-01-13

**Authors:** Laura Pérez-Fernández, Julia Sastre, Carles Zafón, Amelia Oleaga, Esmeralda Castelblanco, Ismael Capel, Juan C. Galofré, Sonsoles Guadalix-Iglesias, Antonio De la Vieja, Garcilaso Riesco-Eizaguirre

**Affiliations:** ^1^ Department of Endocrinology, Hospital del Tajo, Madrid, Spain; ^2^ Department of Endocrinology, Hospital Universitario de Toledo, Toledo, Spain; ^3^ Diabetes and Metabolism Research Unit (VHIR) and Department of Endocrinology, Hospital Vall d’Hebron, Universitat Autónoma de Barcelona, Barcelona, Spain; ^4^ Department of Endocrinology and Nutrition, Basurto University Hospital, Bilbao, Spain; ^5^ Department of Endocrinology and Nutrition, University Hospital Arnau de Vilanova, IRBLLEIDA, Lleida, Spain; ^6^ Endocrinology Department, Parc Taulí Sabadell University Hospital, Sabadell Barcelona, Spain; ^7^ Department of Endocrinology, Instituto de Investigación Sanitaria de Navarra (IdiSNA), Clínica Universidad de Navarra, Pamplona, Spain; ^8^ Department of Endocrinology, Hospital Universitario 12 de Octubre, Madrid, Spain; ^9^ Endocrine Tumor Unit (UFIEC), Instituto de Salud Carlos III (ISCIII), Madrid, Spain; ^10^ Ciber de Cancer (CIBERONC), Instituto de Salud Carlos III (ISCIII), Madrid, Spain; ^11^ Department of Endocrinology and Nutrition, Hospital Universitario de Móstoles, Madrid, Spain; ^12^ Endocrinology Molecular Group, Faculty of Medicine, Universidad Francisco de Vitoria, Madrid, Spain

**Keywords:** dynamic risk stratification, BRAF, multicenter study, thyroid cancer, prognosis, response to therapy

## Abstract

**Introduction:**

The dynamic risk stratification (DRS) is a relatively new system in thyroid cancer that considers the response to primary treatment to improve the initial risk of recurrence. We wanted to validate DRS system in a nationwide multicenter study and explore if the incorporation of BRAFV600E into DRS helps to better categorize and predict outcomes.

**Materials and methods:**

Retrospective study of 685 patients from seven centers between 1991 and 2016, with a mean age of 48 years and a median follow-up time of 45 months (range 23-77). The overall BRAFV600E prevalence was 53.4%. We classified patients into four categories based on DRS (‘excellent’, ‘indeterminate’, ‘biochemical incomplete’, and ‘structural incomplete’ response). Cox regression was used to calculate adjusted hazard ratios (AHR) and proportions of variance explained (PVEs).

**Results:**

We found 21.6% recurrences and 2.3% cancer-related deaths. The proportion of patients that developed recurrence in excellent, indeterminate, biochemical incomplete and structural incomplete response to therapy was 1.8%, 54%, 91.7% and 96.2% respectively. Considering the outcome at the end of the follow up, patients showed no evidence of disease (NED) in 98.2, 52, 33.3 and 25.6% respectively. Patients in the structural incomplete category were the only who died (17.7%). Because they have similar outcomes in terms of NED and survival, we integrated the indeterminate and biochemical incomplete response into one unique category creating the 3-tiered DRS system. The PVEs of the AJCC/TNM staging, ATA risk classification, 4-tiered DRS, and 3-tiered DRS to predict recurrence at five years were 21%, 25%, 57% and 59% respectively. BRAFV600E was significantly associated with biochemical incomplete response (71.1 vs 28.9%) (HR 2.43; 95% CI, 1.21 to 5.23; p=0.016), but not with structural incomplete response or distant metastases. BRAF status slightly changes the AHR values of the DRS categories but is not useful for different risk grouping.

**Conclusions:**

This is the first multicenter study to validate the 4-tiered DRS system. Our results also show that the 3-tiered DRS system, by integrating indeterminate and biochemical incomplete response into one unique category, may simplify response to therapy keeping the system accurate. BRAF status does not provide any additional benefit to DRS.

## Introduction

Papillary thyroid carcinoma (PTC) accounts for approximately 85% of thyroid cancers. Most of the cases are indolent and have a good prognosis and treatment and monitoring methods should depend on the risk of disease progression and clinical evolution. The initial staging systems from the American Thyroid Association (ATA) and the American Joint Cancer Committee/Union Internationale Contre le Cancer (AJCC/UICC) provide valuable information on initial risk estimates for structural recurrence and mortality respectively, which are important for initial treatment and management (1). The dynamic risk stratification system (DRS) proposed by Tuttle in 2008 considers the response to primary treatment during the first two years of follow up and has been shown to modify and improve the initial risk of recurrence (2). This 4-tiered DRS system has been validated in several single center studies, mainly in patients treated with total thyroidectomy (TT) and radioactive iodine therapy (RAI) and its use is strongly recommended in the ATA 2015 guidelines (1–7). There is also evidence that DRS is useful in predicting recurrence in patients treated without RAI (8, 9).

BRAF V600E is the most frequent mutation in PTC (10, 11) and has been related to poor prognosis and disease recurrence (12–15). The ATA guidelines in 2015 suggested to include BRAF status to improve refine the initial risk of structural recurrence in the context of other clinical-pathologic risk factors to aid therapeutic decision-making (1). While BRAF status has been proposed to improve the initial risk stratification of structural recurrence in PTC, the impact on DRS remains unclear. To our knowledge there is only one single center study that explores this relationship (16). In this nation-wide multicenter study promoted by the Spanish Task Force for Thyroid Cancer on behalf of Spanish Society of Endocrinology & Nutrition (SEEN), we wanted to validate DRS system as a valuable tool to predict patients’ clinical outcome and to examine the utility of BRAFV600E mutation in combination with DRS.

## Materials and methods

We retrospectively reviewed the medical records of 685 patients followed-up in seven different tertiary referral centers in Spain between 1991 and 2016 as an initiative Spanish Task Force for Thyroid Cancer. Every center had the approval of their institutional review board. All patients had been treated with total thyroidectomy and 85.5% received additional treatment with RAI. Tumor-nodes-metastases (TNM) staging was standardized post operatively in accordance with the 7^th^ edition of the classification of the Union for International Cancer Control and ATA risk stratification was calculated according to 2015 guidelines (1). Response to primary treatment was evaluated 9-12 months after RAI administration. Stimulated Tg was assessed after either administering recombinant human thyrotrophin (rhTSH) or levothyroxine withdrawal. Long-term follow-up was monitored by clinical examination, neck US and measurement of serum thyroglobulin (Tg) and Tg antibodies (TgAb) concentrations during LT4 treatment to detect recurrent/persistent disease, from now on recurrence. The BRAFV600E mutation status was determined for research purposes in consecutive patients at each center and did not affect decisions on selection of treatments. BRAF status was determined by Sanger sequencing using formalin-fixed-paraffin-embedded tissues in all centers except for one (Clínica Universidad de Navarra) which used pyrosequencing in cytology samples. PTC recurrence was defined as recurrent or persistent disease per authoritative histologic, cytologic, radiographic, or biochemical criteria (1). Local, regional, and distant recurrences were all included. Every investigator was asked to follow the 2015 ATA guidelines and its own clinical expertise. The biochemical criteria for definition of recurrent/persistent disease were abnormal serum Tg levels overtime (suppressed Tg >0.2 ng/ml or stimulated Tg >1 ng/mL) or rising/persistent anti-Tg antibodies TgAb overtime. Follow-up time was defined as the time from initial surgical treatment to the finding of PTC recurrence or, in cases of no recurrence, to the most recent clinic visit.

This was a retrospective study approved by the relevant institutional review boards or ethics committees of each center (Comités de Ética de la Investigación (CEI): CEIm Hospital Universitario 12 de Octubre, CEIm Gerencia de Atención Especializada de Toledo, CEIm del Hospital Universitari Vall d’Hebron, CEI del Hospital de Basurto, CEIm Hospital Universitari Arnau de Vilanova de Lleida, CEIm Parc Taulí and CEIm Clínica Universitaria de Navarra) and was conducted in accordance with the principles of the Declaration of Helsinki and Good Clinical Practice Guidelines. Patients provided written informed consent before study enrolment; patient consent was waived in some cases following institutional review board–approved procedures in the collection of pathological data. The study involved the use of only thyroid tumor tissues and clinicopathological information of patients. The BRAF V600E mutation status was determined after surgical and medical (e.g., radioiodine) treatments in all cases and did not affect decisions on selection of treatments. Genomic DNA isolated from primary PTC tumors was used to analyze the sequence of exon 15 of the BRAF gene for BRAF V600E.

There were four response-to-therapy categories: excellent response, indeterminate response, biochemical incomplete response, and structural incomplete response. These categories were based on the criteria proposed by *Tuttle et al.* in 2010 and adopted by the 2015 ATA guidelines (1, 6, 17). Such criteria have been defined for patients treated with total thyroidectomy and RAI ablation (6). Patients with excellent response are those with negative imaging and either suppressed Tg < 0.2 ng/mL or TSH-stimulated Tg < 1 ng/mL. Patients with indeterminate response to treatment have nonspecific findings on imaging studies, faint uptake in thyroid bed on RAI scanning, a nonstimulated Tg detectable but <1 ng/mL or stimulated Tg detectable but < 10 ng/mL or anti-Tg antibodies stable or declining in the absence of structural or functional disease. A biochemical incomplete response means a negative imaging with suppressed Tg ≥1 ng/mL or TSH-stimulated Tg > 10 ng/mL or rising anti-Tg antibody levels. And finally, a structural incomplete response means structural or functional evidence of disease with any Tg level with or without anti-Tg antibodies. For patients treated only with total thyroidectomy (without RAI ablation) (17) the criteria are a bit different regarding excellent response, indeterminate response, and biochemical incomplete response. Patients with excellent response must have negative imaging and either suppressed Tg < 0.2 ng/mL or TSH-stimulated Tg < 2 ng/mL. Patients with indeterminate response to therapy may have nonspecific findings on imaging studies with a suppressed Tg level of 0.2-5 ng/mL or stimulated Tg level of 2-10 ng/mL or anti-Tg antibodies stable or declining in the absence of structural or functional disease. Finally, patients with biochemical incomplete response to therapy must have a negative imaging and suppressed Tg ≥5 ng/mL or TSH-stimulated Tg > 10 ng/mL or rising anti-Tg antibody levels over time.

We considered patients with no evidence of disease (NED) at the end of the follow up if they had a thyroglobulin less than 0.2 ng/mL under LT4 treatment and less than 1 ng/mL after rhTSH stimulation with negative TgAb and no suspicious images on US neck examination.

We stratified patients by BRAF V600E mutational status and categories of DRS. Cox regression was used to calculate unadjusted and adjusted hazard ratios for risk of recurrence. Recurrence-free survival was calculated by Kaplan-Meier method using log-rank test for comparisons.

To estimate how well a risk stratification system explained recurrence we used the proportions of variance explained (PVEs) in Cox regression models. PVE (%) ranges from 0 to 100, larger numbers mean better predictions. PVE was determined using the mathematical formula: PVE=1-exp(-G^2^/n), where G^2^ is the maximum likelihood ratios that is determined on analysis of chi-squared test associated with the null hypothesis; n is the total number of valid cases in the study (18).

The chi-squared test or Fisher`s exact test was used to compare categories data as appropriate. Hazard ratios (HR) with 95% confidence intervals (95% CI) were calculated in logistic regression model. A two-tailed P value less than 0.05 was considered statistically significant. All the statistical analyses were performed using the statistical package SPSS version 15 for windows and R 4.1 (The R Foundation, Vienna, Austria; http://www.R-project.org/) software.

## Results

### Patient demographics

We studied a total of 685 patients with PTC (540 females and 145 males) across the 7 centers, with an average age of 48. The median follow-up time was 45 months (IQR 23-77). All patients were treated with total thyroidectomy and 85.5% with RAI ablation. Patient age, sex, AJCC/TNM stage, BRAF V600E mutation status, PTC recurrent/persistent disease, follow-up time and provider center are summarized in **Table 1**. In 68.9% of patients, AJCC/TNM classification was stage I or II, the overall BRAF V600E mutation prevalence was 53.4% and the overall PTC recurrent/persistent disease was seen in 21.6% of patients, all of which were comparable with the literature. The dominant PTC histological subtype was classic (68.9%). Following the DRS system, patients were classified into four groups according to the response to initial therapy: excellent (72.6%), indeterminate (8.6%), biochemical incomplete (5.9%), and structural incomplete (12.9%), summarized in **Table 2**.

**Table 1:** Characteristics of 685 PTC patients with known postoperative BRAF status.

	No. of patients	Average Age, y	Male sex, n(%)	Tumor size, Median (mm) [IQR]	Multifocality, n(%)	Classic sub-type, n(%)	TNM stage I-II, n (%)	Radioactive iodine treatment (%)	ATA risk system, n(%)	Follow-up duration, Median (months) [IQR]	BRAFV600, n(%)	Recurrence, n(%)
**Toledo**	195	46	44 (22.6)	13 [8-25]	64 (32.8)	170 (87.2)	147 (75.4)	170 (87.2)	Low 123 (63)	53 [27.5-81]	109 (55.9)	38 (19.5)
									Int. 60 (30.8)			
									High 10 (5.1)			
**Vall d’Hebron**	126	51	27 (21.4)	13.5 [7-25]	60 (47.6)	92 (73)	91 (72.2)	101 (80.2)	Low 76 (60.3)	36 [20-51]	61 (48.4)	24 (19)
									Int. 43 (34.1)			
									High 6 (4.8)			
**Basurto**	99	51	20 (20.2)	13 [10-22]	51 (51.5)	62 (62.6)	68 (68.7)	93 (93.9)	Low 64 (64.6)	27 [17.5-41.5]	46 (46.5)	13 (13.1)
									Int. 35 (35.3)			
									High 0 (0)			
**Arnau**	85	46	15 (17.6)	30 [16-40]	28 (32.9)	59 (69.4)	59 (69.4)	80 (94.1)	Low 58 (68.2)	82 [50-128]	41 (48.2)	28 (32.9)
									Int. 19 (22.3)			
									High 8 (9.4)			
**Parc Tauli**	66	44	12 (18.2)	22 [15-30]	33 (50)	41 (62.1)	43 (65.1)	56 (84.8)	Low 41 (62.1)	78.5 [39.2-157]	33 (50)	26 (39.4)
									Int. 22 (33.3)			
									High 3 (4.5)			
CUN*	63	48	15 (23.8)	13 [10-20]	24 (38)	23 (36.5)	31 (49.2)	49 (77.7)	Low 32 (50.8)	29 [9-56]	45 (71.4)	10 (15.9)
									Int. 23 (36.5)			
									High 1 (1.6)			
**Doce de Octubre**	51	52	12 (23.5)	16.5 [11.5-25]	25 (49)	25 (49)	34 (66.6)	37 (72.5)	Low 21 (41.2)	33 [10-89]	31 (60.8)	9 (17.6)
									Int. 15 (29.4)			
									High 6 (11.8)			
Total	685	48.28	145 (21.1)	15 [10-26]	285 (41.6)	472 (68.9)	473 (69)	586 (85.5)	Low 415 (60.6)	45 [23-77]	366 (53.4)	148 (21.6)
									Int. 217 (31.7)			
									High 34 (4.9)			

*CUN, Clínica Universidad de Navarra.

**Table 2:** Response to therapy categories in 642 patients with collected data.

Medical Center	No. of patients	Excellent, n (%)	Indeterminate, n (%)	Biochemical Incomplete, n (%)	Structural Incomplete, n (%)
**Toledo**	195	148 (75.9)	14 (7.2)	14 (7.2)	19 (9.7)
**Vall d’Hebron**	126	101 (80.2)	2 (1.6)	7 (5.6)	16 (12.7)
**Basurto**	97	81 (83.5)	10 (10.3)	2 (2.1)	4 (4.1)
**Arnau**	76	52 (68.4)	8 (10.5)	2 (2.6)	14 (18.4)
**Parc Tauli**	66	39 (59.1)	3 (4.5)	8 (12.1)	16 (24.2)
**CUN**	54	32 (59.3)	11 (20.4)	3 (5.6)	8 (14.8)
**Doce de Octubre**	28	13 (46.4)	7 (25)	2 (7.1)	6 (21.4)
Total	642	466 (72.6)	55 (8.6)	38 (5.9)	83 (12.9)

### BRAF V600E mutation status, clinical-pathological features and recurrence

The association between BRAF V600E mutation and poor clinical-pathological characteristics and outcome has been well studied (19, 20). Our multicenter study supports an association of BRAF V600E with extrathyroidal extension (68.6% vs. 31.4%; p<0.001) and lymph node metastasis (64.8% vs. 35.2%; p=0.004), but not with distant metastasis (42.9% vs. 57.1%; p=0.163). We also studied the association of BRAF V600E with recurrence in logistic regression analysis. Overall, 25.13% (92 of 366) of BRAF V600E positive patients and 17.55% (56 of 319) of BRAF V600E negative patients experienced recurrence with an unadjusted OR of 1.65 (95% CI, 1.13 to 2.42) (p 0.009), which remained significant after adjustment for patient age and sex (OR, 1.73; 95% CI, 1.19 to 2.56)(p 0.005) yet it did not remain significant after additional adjustment for tumor size, extrathyroidal invasion, lymph node metastasis, multifocality and RAI (OR, 1.57; 95% CI, 0.95-2.62) (p=0.078).

### Validation of dynamic risk stratification

The ability of each of the prognostic system (AJCC, ATA risk stratification and DRS) to predict final clinical status is presented in **Table 3**. As expected, AJCC stage IV patients were more likely to die from thyroid cancer (13.8% vs. 0.5% of stage I). Considering recurrence rate, in AJCC stages I and II, the proportion of patients that have recurrent/persistent disease is relatively high (19.2% and 22%, respectively). When we classified patients according to the ATA recurrence risk classification, the proportion of patients that have recurrent/persistent disease in low, intermediate, and high-risk categories was 10.2%, 38% and 96.8% respectively. Regarding mortality, high-risk patients were more likely to die (36.4%) than patients in the intermediate (0.5%) and the low-risk category (0.8%).

**Table 3:** Final outcomes based on stratification by AJCC, ATA risk of recurrence at diagnosis and DRS.

Persistence/recurrence (%)		Death (%)	
AJCC (n= 594)		AJCC (n= 609)	
I	74/385 (19.2)	I	2/393 (0.5)
II	13/59 (22)	II	0/63 (0)
III	16/87 (18.4)	III	1/88 (1.1)
IV	33/63 (52.4)	IV	9/65 (13.8)
ATA (n= 624)		ATA (n= 639)	
Low	40/393 (10.2)	Low	3/399 (0.8)
Intermediate	76/200 (38)	Intermediate	1/207 (0.5)
High	30/31 (96.8)	High	12/33 (36.4)
DRS (n= 615)		DRS (n= 624)	
Excellent response	8/451 (1.8)	Excellent response	0/457 (0)
Indeterminate response	27/50 (54)	Indeterminate response	0/51 (0)
Biochemical incomplete	33/36 (91.7)	Biochemical incomplete	0/37(0)
Structural incomplete	75/78 (96.2)	Structural incomplete	14/79 (17.7)

When we classified patients based on DRS, the proportion of patients that developed recurrent/persistent disease in excellent, indeterminate, biochemical incomplete and structural incomplete response to therapy was 1.8%, 54%, 91.7% and 96.2% respectively. Patients in the structural incomplete category were the only ones who died (17.7%). Considering the outcome at the end of the follow up (median 3.75 years), patients showed no evidence of disease (NED) in 52%, 33.3% and 25.6% of indeterminate, biochemical incomplete and structural incomplete responders, respectively (**Table 4**). In clinical practice, the DRS (response to therapy re-stratification system) is used to modify initial risk estimates provided by the ATA risk stratification system. As shown in **Table 5**, the change in risk estimates is more evident when an excellent response to therapy is achieved, as it decreases the risk of recurrence/persistence to less than 3% regardless of initial risk estimates. This is especially evident in ATA low and intermediate risk patients. ATA high-risk patients barely had excellent responses.

**Table 4:** Outcomes concerning no evidence of disease (NED) in the different DRS categories.

DRS categories	Persistence/recurrence (%)	NED (%)
**Excellent response**	8/451 (1.8)	443/451 (98.2)
**Indeterminate response**	27/50 (54)	26/50 (52)
**Biochemical incomplete**	33/36 (91.7)	12/36 (33.3)
**Structural incomplete**	75/78 (96.2)	20/78 (25.6)

**Table 5:** Impact of response to initial therapy assessment on initial estimates of risk.

	ATA initial risk of recurrence/persistence classification (n)		
	Low, %	Intermediate, %	High, %
**Initial estimate risk of recurrence/persistence**	10.2 (40/393)	38 (76/200)	96.8 (30/31)
Modified estimate of risk of recurrence/persistence disease based on response to therapy			
**Excellent response**	1.5 (5/336)	2.7 (3/111)	0 (0/1)
**Indeterminate response**	35 (7/20)	65.5 (19/29)	100 (1/1)
**Biochemical incomplete**	91.7 (11/12)	90.5 (19/21)	100 (3/3)
**Structural incomplete**	93.3 (14/15)	94.6 (35/37)	100 (25/25)

Overall, when comparing staging systems, DRS showed more accuracy in predicting recurrent/persistent disease in the lowest stage (**Table 3**). While patients classified as AJCC stage I/II and ATA low risk had persistent/recurrent disease in 19.2%/22% and 10.2% respectively, patients with excellent response had only 1.8%. DRS also showed high accuracy in predicting mortality as patients in the structural incomplete category were the only ones who died (17.7%). On the other hand, in our study, a high proportion of patients in the indeterminate response to therapy category had persistent/recurrent disease, 54%, and 52% had NED at the end of the follow up, which is more than expected.

**Figure 1:**
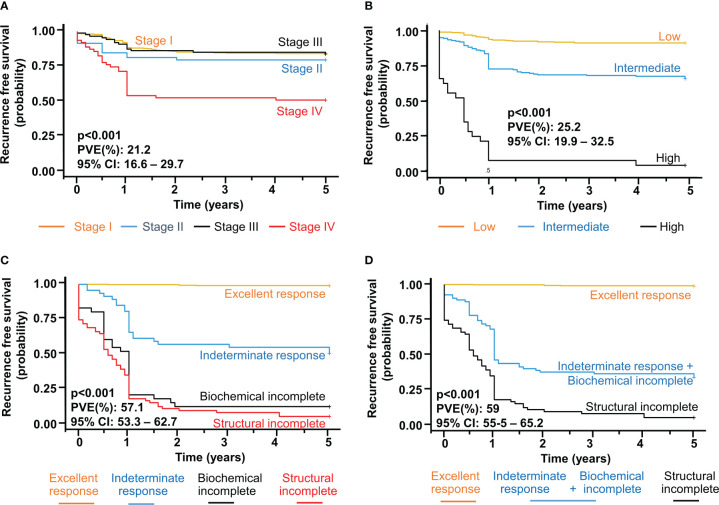
Recurrence Free Survival (RFS) and proportion of variance explained (PVE) to assess the performance of **(A)** seventh AJCC TNM stage, **(B)** ATA risk system, **(C)** 4-tiered dynamic risk stratification (DRS) and **(D)** 3-tiered DRS (integrating indeterminate and biochemical incomplete). Adjusted Hazard Ratios with 95% confidence intervals (95% CI) were calculated in logistic regression model. A two-tailed P value less than 0.05 was considered statistically significant.

Finally, to estimate how well a staging system predicts recurrence we used the proportions of variance explained (PVEs) in Cox regression models. We first calculated the adjusted hazard ratios (AHRs) (adjusted with age at diagnosis, sex, tumor size, extrathyroidal invasion, lymph node metastasis) for risk of recurrence for each system. Because the indeterminate and biochemical incomplete response categories have very similar outcomes in terms of NED and survival, we integrated them into one unique category creating the 3-tiered DRS system. The PVE value of the AJCC/TNM staging, ATA risk classification, 4-tiered DRS, and 3-tiered DRS was 21.2% (95% CI:16.6-29.7), 25.2% (95% CI: 19.9-32.5), 57.1% (95% CI: 53.3-62.7) and 59% (95% CI: 55.2-65.2) respectively (**Figure 1**). Overall, our study supports the 4-tiered DRS as a better system to predict the evolution of patients than classical static systems as the ATA risk classification or the AJCC stages fundamentally at lower stages. In our series, patients in the indeterminate response to therapy have worse outcomes than expected in terms of persistent/recurrent disease and no evidence of disease at the end of follow up. By integrating indeterminate and biochemical incomplete response into one unique category, the 3-tiered DRS system is equally effective in predicting outcomes.

### Association of BRAF V600E with DRS categories

To analyze the impact of BRAF V600E on DRS we first grouped the patients into those with excellent response to therapy at the end of the follow-up and those who did not (non-responders). We found that BRAF V600E patients had a higher probability of having a no excellent response to therapy (31.4%) than the BRAF WT (22.9%) (HR 1.53; 95% CI, 1.080 to 2.188), being this difference statistically significant (p 0.017). Furthermore, within the non-responders, we found that BRAF mutation was specifically associated with the biochemical incomplete response but not with the structural incomplete response. As shown in **Table 6**, 71.1% of patients with a biochemical incomplete response were BRAF positives while only 28.9% were negatives (HR 2.43; 95% CI, 1.21 to 5.23), being this difference statistically significant (p 0.016).

**Table 6:** Impact of BRAFV^6ooE^ in DRS.

Frequencies				Odd ratios		
	BRAF mutated	BRAF wild type	p-value	OR	95% CI	p-value
***DRS***			0.040			
Excellent response	234 (50.2%)	232 (49.8%)				
Indeterminate response	34 (61.8%)	21 (38.2%)		1.61	0.91 - 2.89	0.106
Biochemical incomplete	27 (71.1%)	11 (28.9%)		2.43	1.21 – 5.23	0.016
Structural incomplete	46 (55.4%)	37 (44.6%)		1.23	0.77 – 1.98	0.382

### Refining risk prediction for recurrence using the BRAF mutation

Molecular status, mainly BRAF, can be used to refine risk predictions in combination with ATA risk classification system (1). Likewise, we wanted to explore whether BRAF mutation could refine risk estimates in DRS. We calculated the adjusted hazard ratios (AHRs) (adjusted with age at diagnosis, sex, tumor size, extrathyroidal invasion, lymph node metastasis) for risk of recurrence in each category of DRS with and without BRAF mutation. When patients were stratified according to BRAF mutational status, the AHRs for recurrence increased with increasing DRS severity both in mutant and wild type BRAF (**Table 7**). In the indeterminate category, patients with BRAF mutation show a slightly higher risk than those with BRAF wt during the follow up. Similar trend is seen in the structural incomplete category. However, overall, the differences of the AHRs in each DRS category with and without BRAF mutation (**Figure 1**) are too small for creating different risk grouping and, thus, an integrated prognostic system could not be developed. Thus, BRAF status does not provide any additional benefit in combination with DRS to refine risk predictions.

**Table 7:** Hazard Ratios of categories of dynamic risk stratification for 5 years-recurrence by BRAF V600E mutational.

	BRAF wild type						BRAF V600E					
	Recurrence		Unadjusted		Adjusted		Recurrence		Unadjusted		Adjusted	
Response to therapy	Events/Total (%)	5 y DFS (%)	HR (95% CI)	*P*	HR (95% CI)	*P*	Events/Total (%)	5 y DFS (%)	HR (95% CI)	*P*	HR (95% CI)	*P*
**Excellent**	1/228 (0.4)	229 (100)	—	–	—	–	4/221 (1.8)	221 (98.2)	4.15 (0.46-37.1)	0.203	3.2 (0.36-28.7)	0.299
**Indeterminate**	9/18 (50)	13 (68.4)	160 (20.3-1.263)	<0.001	103 (12.9-823)	<0.001	14/28 (50)	22 (68.8)	156 (20.5-1.189)	<0.001	146 (18.9-1.131)	<0.001
**Biochemical incomplete**	10/10 (100)	6 (60)	798 (101-6.306)	<0.001	647 (75-5.589)	<0.001	21/25 (84)	12 (46.2)	466 (62.4-3.474)	<0.001	477 (61.8-3.676)	<0.001
**Structural incomplete**	33/33 (100)	18 (52.9)	771 (105-5.674)	<0.001	444 (57-3.453)	<0.001	34/37 (91.9)	22 (52.4)	688 (93.6-5.058)	<0.001	652 (84.9-5.013)	<0.001

## Discussion

In this real-world study, we wanted to validate the clinical utility of DRS as compared with the more static AJCC/TNM staging system and the ATA risk-stratification system. We found that DRS is an accurate system to predict the evolution of patients, achieving higher PVEs compared with the two other systems, particularly in excellent responders. We also found the indeterminate and biochemical incomplete response have very similar outcomes in terms of NED and survival at the end of follow up (median 3.75 years). Thus, we integrated them into one unique category creating the 3-tiered DRS system achieving a PVE as good as the 4-tiered. BRAF status does not provide any additional benefit in refining risk estimates when combined with DRS.

All the static staging systems published provide suboptimal long-term predictions for recurrence in individual patients, as demonstrated by the proportion of variance explained (PVE), ranging from 20% to 30% across the studies (a measure of how well a predictive model correlates with the outcome of interest) (17). However, several uncentre studies have demonstrated that when these initial risk estimates are modified over time as a response to therapy, risk estimates can achieve a PVE as high as 70% to 80%. Our study reached a PVE around 60%, comparable to previous results (3, 4, 21). Of note, to calculate the PVE we adjusted for age, tumor size, extrathyroidal invasion, lymph node metastasis, multifocality and iodine treatment, making our findings more robust. However, our results also show that the indeterminate and the biochemical incomplete response to treatment categories have similar outcomes at the end of the follow up in terms of NED and survival compared with the structural incomplete, raising the question whether we could integrate them into one unique category.

The indeterminate response to treatment category, initially described as an acceptable response, was designed for patients with nonspecific findings (including mildly elevated serum Tg) that could not be confidently described as benign or malignant. Over time, 15% to 20% of these patients will develop structural disease that may require additional therapy. In the remainder, the nonspecific changes resolve, and many of these patients can be reclassified as having an excellent response to treatment over time (22). In our study, 54% of the patients in the indeterminate response category developed persistent/recurrent disease and 52% presented NED over time (median 3.75 y), being subsequently reclassified as excellent responders. Likewise, 91.7% of the patients in the biochemical incomplete response to treatment category developed persistent/recurrent disease and 33% presented NED over time (median 3.75 y), being subsequently reclassified as excellent responders. No deaths were observed in the two categories. Although we did not report which patients developed structural disease or needed further treatment, it has been estimated that 15% to 20% of the patients in both indeterminate and biochemical incomplete response will develop structural disease (23). Thus, because the outcome of patients in these two categories are similar, we suggest integrating indeterminate and biochemical incomplete categories into one unique category to simplify response to therapy. We show that the PVE in this 3-tiered DRS system remains high. The patients in this new category will need monitoring and some degree of TSH suppression in the long term to detect and prevent the appearance of structural disease, particularly those that do not achieve NED (between 48% to 67%). We believe that the main factor to establish risk estimates after the initial treatment in these patients would be the levels of Tg overtime (i.e., Tg doubling time) rather than establishing cut-offs that are necessarily arbitrary.

In a similar study by Jeon MI et al. (4), patients with indeterminate and biochemical response reached NED in 74% and 27% respectively after a median of 8 years follow up (in our study, 52% and 33% respectively, 3.75 years follow up). In such study, patients with an undetectable level of serum stimulated Tg (<1 ng/ml), negative TgAb, and no suspicious structural disease at the end of the follow-up period were considered to have NED. These criteria were very similar to ours. We also considered a non-stimulated Tg less than 0.2 ng/mL as another criteria. Thus, stricter criteria to define evidence of disease and a shorter follow up may explain the differences between our study and theirs. Nevertheless, we consider that these criteria adjust better to real clinical practice because detectable levels of thyroglobulin, even if low, normally lead to more frequent blood tests and/or radiological tests, particularly in patients that have undergone a total thyroidectomy and 131-I ablation, in which Tg is most reliable as a marker. In all, considering both studies, the likelihood of achieving NED overtime in the indeterminate category is between 52-74%, which means that some patients will need monitoring for a relatively long period of time and some of them will develop structural disease. From a practical point of view, the clinical management of patients in the indeterminate category would not be very different from the biochemical.

To our knowledge, there is only one previous publication that analyses the relationship between BRAF V600E and DRS (16). This was a retrospective single center study with a total of 723 patients and failed to find a statistical relationship between BRAFV600E and any category of the DRS system. In the mentioned study, the authors found no association between BRAFV600E and cervical lymph nodes metastases, which were only present in the 13% of the patients, which is less than normally seen in the literature. In our study this percentage ascends to 41%, more accordingly to the literature. We found that patients with BRAFV600E have significantly less excellent response to therapy and, particularly, are significantly more likely to develop incomplete biochemical response to treatment. Most of the patients in this category are likely to have microscopic disease affecting cervical lymph nodes or remnants in the thyroid bed that cannot be detected by imaging tests but still produce detectable levels of Tg. Altogether, our results point to BRAFV600E as an oncogene that renders tumors prone to disseminate predominantly in a locoregional manner *via* lymphangiogenesis (24).

In addition, in the indeterminate response to treatment category, patients with BRAF mutation show a slightly higher risk (AHR) than those with BRAF wt during the follow-up and a similar trend is seen in the structural incomplete category. Although it could be of some value whether patients with BRAF mutation in these two categories have more risk in the long term than those without BRAF mutation, the differences are too small to be clinically relevant. Most importantly, the differences of the AHRs in each group of the four-tiered DRS system with and without BRAF mutation (**Figure 1**) are not big enough to create an integrative prognostic system. Thus, it seems that BRAF status does not provide any additional benefit in terms of risk grouping in combination with DRS. By contrast, in the study by Kim et al. (21), the authors did demonstrate that knowing TERT status may help in the reshaping of the risk grouping when combined with DRS. In this regard, TERT is clearly a more robust prognostic marker compared to BRAF.

The multicenter and nation-wide nature of this study are the major strengths as they show the utility of response to therapy in real clinical practice. The participating centers have thyroid cancer units that actively follow contemporary standard practice guidelines in the management of thyroid cancer, minimizing the heterogeneity in the management of thyroid cancer. The fact that the overall analysis reproduced the findings seen in the literature regarding the association of BRAF status with aggressive clinico-pathological features is consistent with the good generalizability of the current study (20). On the other hand, this study has some limitations. First, the multicenter and retrospective nature is, nevertheless, inherently associated with the potential limitation of data heterogeneity. The effects of variable confounding factors such as surgical skills, completeness of surgery, strategies of RAI ablation and level of care should be taken into consideration. However, despite this heterogeneity, our study reflects the good performance of DRS across the country, making it more reliable. Similarly, in a recent large multicenter study, the ATA risk stratification system has been shown to be a reliable predictor of short-term outcomes in patients with differentiated thyroid cancer in real-world clinical settings characterized by center heterogeneity (25). Second, the relatively short follow-up period (average 5 years) is also a limitation as we cannot rule out that our results may change in a longer period.

To conclude, in this nation-wide multicenter study we provide evidence to support the 4-tiered DRS as a better system to predict the evolution of patients compared to AJCC/TNM staging system and the ATA risk-stratification system. However, our results also show that the indeterminate and biochemical incomplete response categories have similar outcomes in terms of NED and survival. Integrating them into one unique category, the subsequent 3-tiered DRS system appears to be equally accurate compared to the 4-tiered DRS, which we believe simplifies the system and makes it more helpful for clinicians. We believe that rather than relying on Tg cutoffs that are somehow arbitrary, watching Tg overtime (i.e., Tg doubling time) would be more helpful in this new category. Finally, in contrast to TERT, BRAF does not provide any additional benefit in terms of risk grouping when combined with DRS.

## Data availability statement

The raw data supporting the conclusions of this article will be made available by the authors, without undue reservation.

## Ethics statement

The studies involving human participants were reviewed and approved by CEIm Hospital Universitario 12 de Octubre, CEIm Gerencia de Atención Especializada de Toledo, CEIm del Hospital Universitari Vall d’Hebron, CEI del Hospital de Basurto, CEIm Hospital Universitari Arnau de Vilanova de Lleida, CEIm Parc Taulí and CEIm Clínica Universitaria de Navarra. The patients/participants provided their written informed consent to participate in this study.

## Author contributions

Conception and design: G.R-E Financial support: GR-E and AV. Administrative support: GR-E. Provision of study materials or patients: JS, CZ, AO, EC, JG, IC, SG-I. Collection and assembly of data: All authors. Data analysis and interpretation: GR-E, AV and LP-F. Manuscript writing: GR-E and LP-F. All authors contributed to the article and approved the submitted version.
